# A holistic approach to psychological sexual problems in women with diabetic husbands

**Published:** 2014-03

**Authors:** Anahita Khodabakhshi Koolaee, Edalat Asadi, Ladan Mansoor, Leili Mosalanejad, Ali FathAbadi

**Affiliations:** 1 *Department of Family Counseling, University of Social Welfare and Rehabilitation Science, Tehran, Iran.*; 2 *Department of Family Counseling, Shahid Beheshti University, Tehran, Iran.*; 3 *Department of Mental Health, Jahrom University of Medical Sciences, Jahrom, Iran.*; 4 *Department of Psychology, Shahid Beheshti University, Tehran, Iran.*

**Keywords:** *Sexual dysfunction*, *Diabetes*, *Sexual activity*

## Abstract

**Background: **One of negative and influential factor to chronic diseases is creation of sexual problems in the couple's sexual relationship. Sexual health is one of the most important factor in Sexual and marital satisfaction.

**Objective:** This study aims to compare the relationship between couple burnout, sexual assertiveness, and sexual dysfunctional beliefs in women with diabetic and non-diabetic husbands.

**Materials and Methods: **This cross sectional descriptive study was a causal comparative one. The research plan was derived from the master’s dissertation for family counseling in Shahid Beheshti University which was done during 2011-2012 in Tehran, Iran. Totally 200 participants were included in this study; 100 participants were women with diabetic husbands and the others were women with non-diabetic husbands. These participants were selected by purposeful sampling method. Data were collected using personality traits and demographic characteristics’ questionnaire, couple burnout Measure, Hulbert index of sexual assertiveness and sexual dysfunctional beliefs Questionnaire.

**Results: **Results of the multi-variable analysis of variance indicated that there was a significant difference between couple burnout, sexual assertiveness, and sexual dysfunctional beliefs in women with diabetic and non-diabetic husbands. Women with non-diabetic husbands had a higher mean score in sexual assertiveness factor compared to women with diabetic husbands, whereas in couple burnout and sexual dysfunctional beliefs factors, women with diabetic husbands had a higher mean score.

**Conclusion:** It seems that one of the most important factors which influences and increases couple burnout, increases wrong sexual beliefs regarding sexual function, and decreases sexual assertiveness in women is their husbands’ sexual dysfunction. In fact, women whose husbands suffer from chronic diseases such as diabetes (which results in sexual dysfunction) have lower sexual assertiveness and higher couple burnout and sexual dysfunctional beliefs compared with other women.

This article is extracted from student thesis. (Edalat Asadi)

## Introduction

The systematic view indicates that an active interaction exists between different types of relationship between human beings and physical and mental health (1). Sexual health in the family is one of the most important issues in mental health. Sexual problems can be discussed by three components including couple burnout, lack of sexual assertiveness, and sexual dysfunctional beliefs. Couple burnout refers to a state of physical, emotional, and mental exhaustion as well as depersonalization and reduced personal accomplishment (2).

Sexual assertiveness is considered as one's capability to make sexual relationships for removing sexual needs and initiating sexual behavior with a partner or spouse, and sexual beliefs are defined as one's thoughts and ideas about sexual behaviors (3, 4). Most men and women feel that sexual relations make no sense without love. They do not like sexual relationships as a sole physiologic activity; instead they like it to be a symbol of love with a spiritual meaning (5). Disenchantment, physical decrepitude and exhaustion are mental and emotional problems caused by wrong expectations and depend on the adjustment of couples and their beliefs (6). Couple burnout sometimes appears due to life vicissitudes and a set of unrealistic expectations (7). 

According to cognitive theory, people form some beliefs about their sexual identity and capabilities. Coffle and Hyman state that beliefs and cognitive schema form the base of behaviors and past experiences (8). The effect of beliefs and myths on sexual affairs has been focused in clinical research for a long period (9). Past studies showed that different sexual beliefs play an important role in shaping sexual dysfunction (4). In men dominated societies, women's sexual tendency is often faced with insult and disgrace and men believe that women are sexual objects who have no sexual needs or rights (10). 

Sexual assertiveness is made by understanding women and men's desires and proper famine and masculine behaviors (11). In many societies, women have problem in asserting themselves and have a low self-esteem and it is difficult for them to express their needs or keep their individual dependency in marital relationships (12). Despite the sexual revolution of this era, women are still followers, from this point of view, as compared with men and most of them believe that in sexual relationships men should be pioneers (13). Also results indicated a strong correlation between sexual self-esteem, sexual assertiveness and sexual satisfaction (9). 

Sexual self-esteem and assertiveness low are among personal characteristics of women who have experienced sexual force in their marital relationships (14). This can be one of the preparing factors for women's sexual disenchantment in marital relationships which is followed by their total couple burnout (15). Evidence revealed that chronic diseases affect sexual health (16). Currently, more than 5.5 million people have diabetes in Iran. One of the long-term effects of this disease is nervous system complications which include sexual dysfunction (17). For negative effects of diabetes mellitus and other chronic diseases on sexual health and performance, we can refer to other studies (18-21). There is a negative and reverse relationship between marital quality and diabetes in couples in which one of the spouses suffered diabetes (18). 

A desirable sexual relationship which can satisfy both sides has an important role in families’ success and stability (12). According to promotion of psychological research on mental health and considering the fact that many of the family sexual problems are not reported in our community due to cultural limitations, it may induce many problems that threaten family mental health. Also several studies are done about sexual problems in diabetic patients, but none of them have investigated sexual problems of women with diabetic husbands. This study aims to survey the relationship between couple burnout, sexual dysfunctional beliefs and sexual assertiveness in women with diabetic husbands and non-diabetic husbands.

## Materials and methods

This cross sectional study was a causal comparative one. The research plan was derived from the Master’s dissertation for family counseling in Shahid Beheshti University which was done during 2011-2012 in Tehran, Iran. 

Study participants were divided into two groups: the first group included women with diabetic husbands who were also members of the central branch of Iranian Diabetes Society- Tehran branch (this Society has different branches in a number of cities) and the second group included women with non-diabetic husbands. 

Purposive sampling method was used in this research. Iranian Diabetic Society (IDS)- Tehran branch declared that 20.000 male and females have registered in this Society. Inclusion criteria were: 1) women with diabetic husband who had registered in Iranian Diabetic Society- Tehran branch, 2) women who had at least finished high school and were able to answer the questions 3) participants who had middle class scio-economic status, 4) women who had two children 5) those who had married 10-20 years ago 6) their age was between 30-40 years old, and 7) having diabetic husbands or husbands under medical treatment of diabetes. 

Exclusion criteria were: 1) illiterate or low educated participants 2) participants who had no child or one child 3) marriage duration was less than 10 years or more than 20 years, 4) having a husband with other physical and mental disorders and 5) patients who had no records or registration number in IDS. From 20.000 diabetic patients who registered in IDS 1500 were included in our study and 135 were interested in collaboration in the study. After the first orientation session, 35 women announced that they had changed their idea and they wanted to participate in the study. Then 100 married women with diabetic husbands and 100 with non-diabetic husbands were randomly selected by researchers. 

100 women with non-diabetic husband who were selected and matched with the target group by age, scio-economic status, level of education, number of children and marriage duration. All the questionnaires were filled out by interviewers and women answered them independently. Before the implementing the research the interviewers were participated in orientation session and were explained them about how to gathering data and to be sensitive about ethical issues. All of interviewers were undergraduate student of Master of Art of family counseling. 

**Research tools**

**Demographic questionnaire (personal traits): **This questionnaire was made by the researchers to assess personal characteristics of participants. This questionnaire included age, husband's age, both the participant and her husband's level of education, couple's age difference and marriage duration. 

**Halbert indicator of sexual assertiveness (HISA): **This questionnaire was provided by Halbert in order to measure women's sexual assertiveness in interaction with others (24). The questionnaire included 25 questions and 5 point Likert scale that was used for its choices. The choices ranked from "always" to “never”; “always=4, often=3, sometimes=2, rarely=1, and never=0”. This questionnaire was normalized in Iranian society. The alpha index was yielded 0.79 for the whole test. In Iran, Sanaee reported the content validity Index to be 0.91 (23). 

**Sexual dysfunctional beliefs questionnaire (SDBQ): **This questionnaire was presented by Nobreh and Pinto-Guya (9). It is a 40-item questionnaire which evaluates sexual beliefs and imaginations that are considered as the preparing factors causing sexual dysfunction in men and women in clinical literature. The questionnaire has been offered in two versions for men and women, which measures beliefs related to each gender specifically. Individuals were asked to announce their agreement in a five point Likert scale ranked from completely disagree=1, disagree= 2, neither agree nor disagree= 3, agree= 4, and strongly agree=5 (9). The subscales of women form were included 6 beliefs= beliefs about woman's X, beliefs about sexuality as a sin, beliefs about body image, beliefs about the priority of emotion upon sexuality and beliefs related to the priority of motherhood duties up on sexual relationship. 

The reliability of test- retest for men and women versions with a four weeks interval, had a acceptable correlation (r=0.73, r=0.80). Chronbach's alpha value for men and women versions was reported to be 0.93 and 0.81, respectively which approved the questionnaire's internal consistency. In the Persian version of this questionnaire internal consistency was calculated for men and women to be 0.89 and 0.80, respectively. The reliability of its convergence with the questionnaire of insufficient attitudes was also 0.76. Also the content validity of consistency reported by creators of SDBQ 0.76 was (9). 

**Couple burnout measure (CBM): **This questionnaire is a self-measure tool designed for measuring couple burnout. This questionnaire includes 21 items which indicate burnout symptoms and has three principle parts: physical (Collapse e.g. feeling tired, weakness and sleep disorders), emotional collapse (e.g. feeling depressed, despair, feeling trapped) and mental collapse (e.g. feeling worthless, frustration and showing anger to spouse). 

All these items are responded on a 7 point scale “Always= 7, often=6, usually= 5, sometimes= 4, rarely= 3, once= 2, and never= 1” (24). In Iran, Navid measured Chronbach’s alpha value for this questionnaire that was 0.86 (24). In Adibrad and Adibrad study, the p-value of test- retest for one month, two months and four months period was 0.89, 0.76 and 0.66, respectively. The internal consistency for most respondents was measured by alpha index which ranged from 0.91-0.93 and content validity was claimed 0.82 (25, 26). All the questionnaires were filled by participants. The proposal extracted from this paper was confirmed by Ethics Committee of Shahid Beheshti University.

**Statistical analysis**

Data analysis was done using descriptive statistics, frequency, percentage, mean, variance and standard deviation. Also analytic statistical methods were used such as variance analyses (ANOVA) and LSD. The confidence level was identified p<0.05.

## Results

Descriptive indicators of demographic characteristic of women with diabetic husbands are presented in table I. The mean age of women with non-diabetic husbands and their spouses were 37.12 and 41.96 3.21±2.28 respectively and the age interval between spouses was 4.73±0.3 years old. Marriage duration was 10.89±1.2. Totally educational level in participants was 12 (12%) diploma, 70 (70%) undergraduate and 18 (18%) post graduate. Education levels of spouse’s were 44 (22%) diploma, 84 (42%) undergraduate and 72 (36%) post graduate. As it is inferred from these data the mean for couples burnout (90.6±10.4) and sexual dysfunctional beliefs (17.15±12.4) in women with diabetic husband was higher and the mean for sexual assertiveness (31.49±17.78) was lower than women with non-diabetic husbands (Table II). As it is resulted from the above table; the value of the statistical indicator (F=3.455) in each four indicators in a significant level of α=0.05. In other words, variance analysis results showed that the there is a difference between couples burnout, sexual dysfunctional beliefs and sexual assertiveness in one of the components of this variable. The results are presented in (Table II). 

After comparing couple burnout, sexual dysfunctional beliefs and sexual assertiveness in women with diabetic and non-diabetic husbands, LSD test was used to reveal the difference between the two groups since F-test results only showed an overall difference and do not determine whether the difference is beneficial for one of the groups. Results of the LSD Test confirmed the difference between sexual problems, couple burnout, and sexual dysfunction scores between women with diabetic and non-diabetic husbands (Table III). Women with non-diabetic spouses had experienced a higher level of sexual assertiveness than women with diabetic husbands. While, women with diabetic husbands had higher levels of couple burnout and sexual dysfunction beliefs (Table IV).

**Table I:** Mean scores and standard deviation of personal characteristics of participants

**Variables**		**Mean**	**SD**
Women with diabetic husbands			
	Age	35.91	4.34
	Spouse's age	44.66	5.11
	Age differences	5.24	1.97
	Marriage duration	13.49	4.14
Women with non-diabetic husbands			
	Age	36.12	4.91
	Spouse's age	43.96	5.09
	Age differences	4.94	2.01
	Marriage duration	13.20	4.11

All differences between groups NS (p>0.05).

P-Value age (p: 0.523)

Spouse's age (p: 0.52)

Age differences (p: 0.60)

Marriage duration (p: 0.56)

**Table II:** The mean and standard deviation of research variables (CBM, HISH & SDBQ) of all participants in study

**Tests**	**Variable**	**Women with diabetic husbands** **(N= 100)**		**Women with non-diabetic husbands** **(N= 100)**		**p-value**
		**Mean**	**SD**	**Mean**	**SD**	
CBM						0.01*
	Couple burnout	90.6	10.4	85.81	12.95	
HISA						0.01*
	Sexual assertiveness	31.49	17.78	37.82	16.95	
	Sexual cautiousness	33.6	5.43	31.87	5.02	
	Sexual tendency	18.66	4.58	17.21	4.01	
SDBQ						0.01*
	Age related beliefs	16.86	3.40	17.06	3.57	
	Sexual self-thought	13.32	3.26	12.75	2.44	
	Denying emotion's priority	21.01	21.21	2.10	5.703	
	Motherhood priority	13.50	1.41	13.50	1.41	
	Sexual dysfunctional beliefs	117.15	12.04	113.40	12.99	

**Table III:** ANCOVA test of mean scores of CBM, HISA and SDBQ questionnaires

**State**	**sources of change**	**SS**	**DF**	**MS**	**F-test**	**p-value**
Group						
	Sexual assertiveness	2003.445	1	2003.445	6.639	0.011
	Couple burnout	1147.205	1	1147.205	8.313	
	Sexual dysfunctional beliefs	703.125	1	703.125	4.479	
Error						0.004
	Sexual assertiveness	59751.75	198	301.777	-	
	Couple burnout	27325.39	198	138.007	-	
	Sexual dysfunctional beliefs	31082.75	198	158.984	-	
Total						0.036
	Sexual assertiveness	301949	200	-	-	
	Couple burnout	1584497	200	-	-	
	Sexual dysfunctional beliefs	2689451	200	-	-	

**Table IV:** LSD test of mean scores of CBM, HISA, and SDBQ questionnaires

**Variable **		**Mean difference (I-J)** *	**SD**	** (p-value)**
Sexual assertiveness				
	Sexual cautiousness	6.33	2.457	0.011
	Sexual tendency	-6.33	2.457	0.011
Couple burnout				
	Age related beliefs	-4.79	1.661	0.004
	Sexual self-thought	4.79	1.661	0.004
	Denying emotion's priority	-3.75	1.772	0.036
	Motherhood priority	3.75	1.772	0.036
Sexual dysfunctional beliefs				

* I-J Non diabetic group- diabetic group.

## Discussion

This study aimed to survey the relationship between couple burnout, sexual dysfunction beliefs and sexual assertiveness in women with diabetic and non-diabetic husbands. As a result, the mean score for couples burnout and sexual dysfunctional beliefs in women with diabetic husband was higher while the mean score for sexual assertiveness was lower than women with non-diabetic husbands. 

One's knowledge about his/her spouse's illness can cause a type of mental disorder since diabetes is a chronic disease, accompanied by several effects and mental disorders, which influences the patient and his/her caregivers more than other chronic diseases. Also it requires a difficult daily management for controlling the disease (27). 

Low level of education, longer duration of diabetes, and poor controlled diabetes were associated with Sexual dysfunction in diabetic patients (28). Sexual problems are related with distress and reduced quality of life. Because of the irreversible nature of sexual problems in chronic illnesses, it is more important to diagnose these problems and understand cognitive and behavioral coping processes in such patients (27, 29). This, in turn, can cause an additional burden of responsibility and family commitment for the diabetic patient's spouse (Family commitments bring about the feeling that his/her emotional aspect has been neglected). On the other hand, one of the long-term effects of diabetes is nervous problems and sexual disorders (30). 

These results confirm our results about diabetic sexual problems among couples. Also, in diabetes, in addition to physical and sexual effects, mental disorders like depression, anxiety and impatience, patient's quality of life especially married life will be subjected to change (31-33). These results confirm our results about sexual problems in couple with diabetes. It is clear that the consequences of this disease can make married life of a diabetic person more vulnerable and result in conjugal dissatisfaction (especially sexual dissatisfaction) in the patient's spouse. 

Low conjugal satisfaction can result in frustration and disenchantment in different aspects of conjugal life (especially sexual relationship) in the spouse of a diabetic person and remarkably reduce his/her quality of life (6). This was also confirmed by present results. The association between weak sexual relationship and couple burnout can have two concepts: when sexual relationship is weak or is not satisfying, couple burnout is facilitated; or burnout causes sexual disorders. In fact, a defective cycle may be formed between these two items. According to couple therapists, weak sexual relationship is the start of destroying conjugal relationships (33, 34). 

The depression and anxiety resulted from diabetes can cause a monotonous and tiring atmosphere and partly take the spirit of happiness joy and variety from conjugal life (32). Some of these results confirmed our related causes of sexual problems in diabetic couples. Also evidence has been showed that patients with chronic illnesses may become uninterested in sexual activity because of misconceptions about their ability in sexuality, body-image concerns or grief related to their disease) 22, 29). 

According to present situation, it is more probable that the spouse of a diabetic person use reverse reaction mechanism under wrong gender and sexual beliefs which roots in the culture of the society and continues to take care of her husband more and more and suppress her demands, tendencies, and conjugal rights generally and sexual assertiveness specifically. 

Of course there are several social and cultural reasons why women may stop their desires and tendencies including sexual ones in their married lives (1). The fact that women believe a stable relationship with companionship and support is more valuable than sexual satisfaction refers to their level of sexual assertiveness. An implication is that women sacrifice sexual satisfaction for the sake of stability and continuity of security. Therefore, the fact is that women who define themselves based on gender and sexual clichés that stem from society's manners cannot express a real sexual ego. This is a beginning for forming a non-active sexual tendency in women (6). 

These results confirm our results about lack of sexual assertiveness in women with diabetic husbands. Hence, it can be said that the wife of a diabetic person, like every other woman, not only indicates unmet demands and expectations in her relationship but also causes more problems, responsibilities and sexual and emotional shortages than other women. In such a situation, she gradually suffers a crisis called couple burnout (30). It is not surprising that such a person will be affected by physical erosion resulted from couple burnout. Mental health problems are accompanied by signs like lack of self-confidence (self-belief), spousal negative comments, despair and self-dissatisfaction and lack of loving one's self (6). 

Results above agree with our results about sexual dysfunction belief in women with diabetic husbands. Sexual problems among women associations with some demographic, socio-economic factors work situation, life-style and health factors (34). This issue becomes of great importance when such problems are hidden or underestimated due to cultural background. 

Therefore it needs for pay attention to this subject in these patients. To done this study researchers were encountered to some obstacles and problems. These issues were faced our research findings with limitations. First of all people’s resistance and sensitiveness to fill out the questionnaire, invading individuals' privacy, the need for a private place participants’ right to be justified about the research. Second, little studies about this subject for the review can be stated as the limitations of this study. The last but not least, our sample was selected from Iranian diabetic association branch of Tehran so, to be generalizing the research findings to other cities of Iran and other minorities must be doing with cautiously.

## Conclusion

In fact, women whose husbands suffer from chronic diseases such as diabetes (which results in sexual dysfunction in patients) have lower sexual assertiveness and higher couple burnout and sexual dysfunctional beliefs compared with other women. Therefore, it is important to pay close attention to such issues because of the importance of family health.
